# The menstrual cycle minimally affects cardiorespiratory function and body balance control in trained women during acute hypobaric hypoxia exposure (4000 m)

**DOI:** 10.1007/s00421-025-05770-w

**Published:** 2025-04-30

**Authors:** Cristina Rotllan, Jan Hagenaers, Marc Colls, Ginés Viscor

**Affiliations:** Facultat de Biologia, Physiology Section, Department of Cell Biology, Physiology and Immunology, Universitat de Barcelona, Barcelona, Spain

**Keywords:** Female athlete, Sports performance, Menstrual cycle, High altitude, Body balance control, Spirometry, Submaximal exercise

## Abstract

**Purpose:**

The impact of the menstrual cycle (MC) on physical performance has been a hot topic in recent years, due to the increasing professionalisation of women’s sports. This study set out to investigate the impact of the MC on the performance of women athletes in a hypoxic environment. The study focused on cardiovascular responses, respiratory function, and postural control as key indicators of sports performance.

**Methods:**

We measured the physiological responses in 20 participants under hypobaric hypoxia (HH) conditions, on two occasions: during the early follicular phase (F) and in the mid-luteal phase (L). Spirometry and postural control parameters at rest were evaluated at sea level and 4000 m simulated altitude in a hypobaric chamber. The exercise performed at hypoxia was divided into two phases of different workload intensities: 40% and 70% of their theoretical maximal rate of oxygen consumption (${\dot{\text{V}}\text{O}}_{{2}} {\text{max}}$), followed by a recovery period.

**Results:**

Tidal volume (VT) significantly decreased at the L phase compared to the F during high intensity exercise (1.69 vs. 1.82, *p* ≤ 0.05) and arterial oxygen saturation (SaO_2_) was greater in the L phase versus F independently of the exercise intensity (*p* ≤ 0.01) under hypoxic conditions. Meanwhile, spirometry and other cardiorespiratory responses did not change across the MC. Lateral velocity increased in the L phase with eyes open compared to F (4.88 vs. 4.24, *p* ≤ 0.05). No significant differences in the performance metrics evaluated between the menstrual cycle phases or between exercise intensities were detected.

**Conclusion:**

Our data confirms that the menstrual cycle in women does not generate sufficiently marked alterations to affect sports performance when acutely exposed to high altitudes.

## Introduction

The number of women practising and competing in sports has increased in the last decades, not only at low-, but also at high-altitude conditions under hypobaric hypoxia (HH) (Horakova et al. 2023). However, the underrepresentation of women in sports science research demands immediate action to delve into female physiology responses during exercise (Costello et al. 2014), to let female athletes achieve their real performance. Ovarian hormones fluctuate cyclically across the menstrual cycle during the fertile years. The follicular phase (F) is the first part of the cycle from the menses, where hormones are at their lowest level to ovulation characterised by high levels of oestrogens. The second part starts after ovulation; oestrogens and progesterone rise progressively until their peak in the mid-luteal phase (L), followed by a decrease in hormone levels until the MC finish the day before bleeding (Elliott-Sale et al. 2021). Despite the scarcity of literature on this topic, in the last two decades, there has been an increase in interest in understanding the potential effect of MC on athletic performance, metabolism, thermoregulation, injuries and recovery (Lei et al. 2019; Romero-Parra et al. 2020; Martin et al. 2021; Barba-Moreno et al. 2022; Williams et al. 2023; Smith et al. 2024). However, research investigating physiological responses to hypoxia across the MC is poorly understood (Raberin et al. 2024).

Exposure to exercise under hypoxic conditions is doubly stressed and performance is limited due to acclimatisation capacity (Ulrich et al. 2017). A decrease in maximal oxygen consumption at different altitudes has been associated with a drop in aerobic performance (West et al. 1983; Wehrlin and Hallén 2006) and similar findings in female runners (Woorons et al. 2005). Upon respiratory system stress during hypoxic exercise at 5,050 m, diaphragm fatigue ensues (Cibella et al. 1996) along with a decrease in arterial oxygen saturation (SaO_2_) (Woorons et al. 2005). Furthermore, the cardiovascular system also plays a central role in hypoxic adaptation, with increased cardiac output and reduced blood transit time at pulmonary capillaries, thus limiting alveolar–arterial diffusion (Ulrich et al. 2017).

Given the established significance of the cardiovascular and respiratory systems on hypoxia adaptation during exercise, the potential impact of female hormones on these systems is a subject of interest. It was hypothesised that oestrogens could improve aerobic performance due to the potential vasodilator effect on the central and peripheral vascular system, boosting blood flow in the muscles and heart, via nitric oxide signalling (Lebrun et al. 2013). Progesterone, the principal hormone in the L phase, has been defined as a regulator of the respiratory function, increasing the respiratory frequency (Rf), yet the precise mechanism is already unknown (Behan and Wenninger 2008). One way progesterone may act is by changing the sensitivity of hypothalamic receptors, thereby reducing the threshold of the respiratory centre and increasing ventilation (${\dot{\text{V}}\text{E}}$) (Rael et al. 2021). It has previously been described how progesterone increases body temperature and its role in thermoregulation (Janse de Jonge 2003). Higher ${\dot{\text{V}}\text{E}}$, heart rate (HR) and tidal volume (VT) at different exercise intensities in the L phase were also found (Rael et al. 2021; Barba-Moreno et al. 2022). In addition, the role of sex hormones in the muscle, bone, ligament and nervous system could impact postural balance and increase injury risk during the L phase (Möller-Nielsen and Hammar 1989).

Regarding the hypoxic ventilatory response (HVR), studies are scarce and often employ different methodologies. In a large cohort study, 336 premenopausal women at 4000 m had significant HVR responses during the L phase, with greater SaO_2_, but not in the hypoxic cardiac response (HCR) (Richalet et al. 2020). Contradictory findings with no changes across the MC were also found (Takase et al. 2002).

The main objective of the present study was to assess the effects of MC on cardiorespiratory and balance response at HH, at rest and during hypoxic exercise at two different intensities in 20 eumenorrheic female cyclists and runners. Furthermore, we hypothesised that subtle differences in physical capacity due to variations in the menstrual cycle, which are not noticeable at sea level, could become evident under simulated altitude conditions. We selected well-trained women of a wide range of ages based on the hypothesis that the high levels of circulating oestrogens and progesterone during the L phase could impact physical performance by increasing aerobic capacity. We also tested the hypothesis that acute hypoxia exposure could affect body balance differently along the MC.

## Methods

### Participants

A total of 20 eumenorrheic women, aged between 18 and 49 years, were included in this study (Table 1). To be eligible for participation, women were required to have a regular MC, (length between 21 and 36 days and no menstrual irregularities) and to have refrained from using hormonal contraceptive treatments for at least 3 months before recruitment. Additionally, all participants were required to be in good health and to have undergone a minimum of 150 min of aerobic exercise/week. Prior to their involvement in the study, all participants were informed about the tests included in the protocol and were required to sign a written consent form. The study was carried out according to the Declaration of Helsinki for human experimentation and approved by the Institutional Ethical Committee from the University of Barcelona (IRB 00003099; CER022441).

**Table 1:** Anthropometric characteristics of the participants (*n* = 20)

	Mean ± SD	Range
Age (years)	33.95 ± 9.11	18–47
Body weight (kg)	56.48 ± 5.21	48–67
Height (cm)	166.4 ± 5.87	157–178
BMI	20.41 ± 1.71	17.86–23.33

*BMI* body mass index

### Menstrual cycle determination

To determine the phases of the MC during the assay, the participants recorded their menstruations in a FitrWoman app (FitrWoman Ltd., n.d.). They also performed urine LH tests (LH60 Ovulation Test Strips 8 MomMed kit) daily from day 7 of the cycle until a positive result was obtained. The urine tests were conducted in the morning with the first urine sample or in some cases, at midday, approximately 3–4 h after the last urine sample. The participants were requested to send a photograph of the test result daily to one of the researchers. The MC phases were determined based on the provided cycle data. The MC phases studied were phase 1, the early follicular phase (F), which corresponds to the initial 5 days of menstruation, and phase 4, defined as the mid-luteal phase (L), which occurs between days 7 and 9 following a positive ovulation test. However, the MC phase determination in this way has some limitations. Serum analysis was not performed and hormonal status could not be accurately determined (Elliott-Sale et al. 2021). The MC characteristics are shown in Table 2.

**Table 2:** Menstrual cycle characteristics (*n* = 20)

	Mean ± SD	Minim	Maxim
Follicular phase length (nº days)	14.43 ± 3.05	9	18
Luteal phase length (nº days)	14.3 ± 3.66	9	26
Ovulation day	14 ± 2.12	9	17

### Acute hypobaric hypoxia

Spirometry, body balance and submaximal two-phase exercise tests were conducted in a hypobaric chamber (Moelco Técnicas Aplicadas SL; Terrassa, Spain) decompressed to the barometric pressure equivalent of 4000 m of altitude (616 hPa). All the hypobaric exposures were completed within 40–90 min, depending on the number of participants evaluated in the same hypobaric exposure trial.

### Study design

**Fig. 1:**
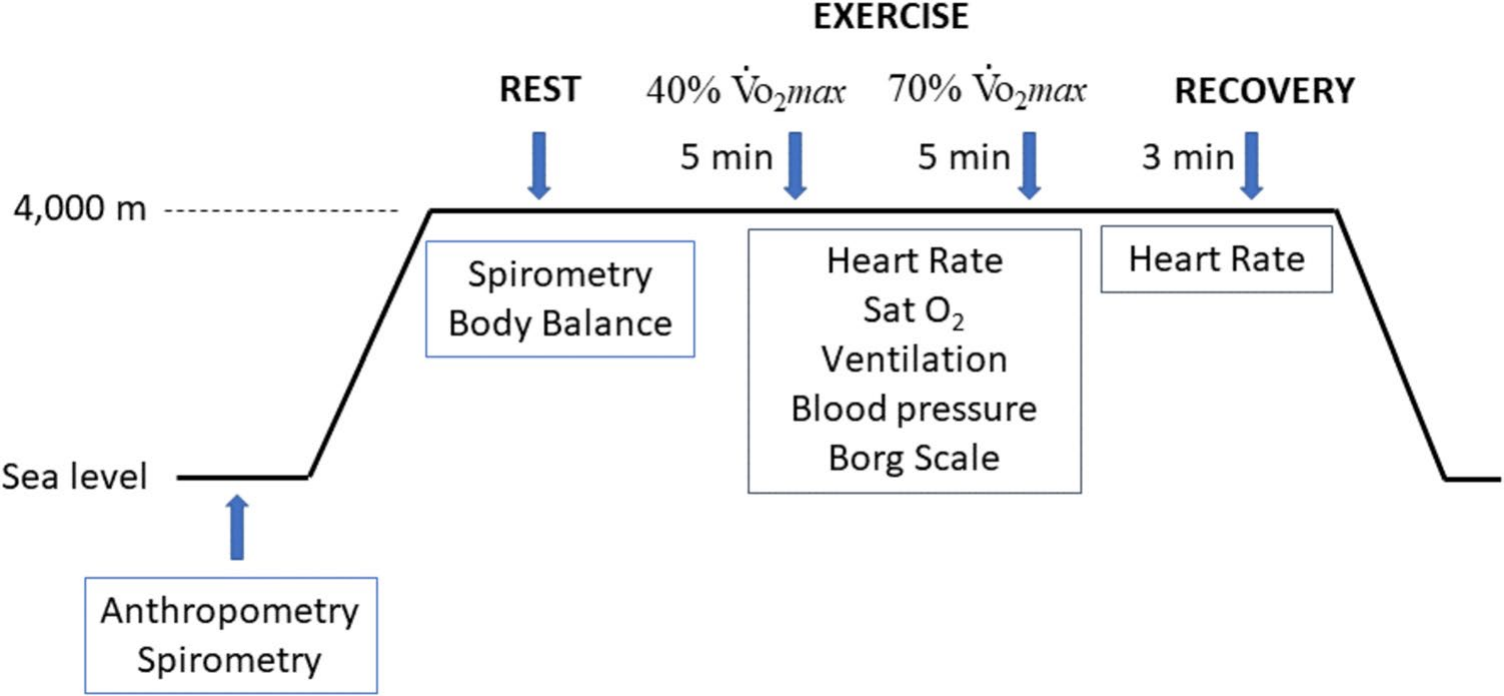
Study design

Participants were referred to the Service of Hypobaria and Biomedical Physiology of the University of Barcelona. Measurements were made under normobaric conditions at sea level (SL) and a simulated altitude equivalent to 4,000 m in the hypobaric chamber (HH). The experimental protocol was repeated twice in two MC phases (F and L). The first visit was performed at a randomly selected phase of the MC, and their body weight and height were recorded. The design of the study is shown in Fig. 1.

Submaximal exercise. The exercise protocol was performed cycling under HH (Bike ERG, Concept2®; Morrisville, EUA) starting with a 3-min warm-up (< 40% theoretical ${\dot{\text{V}}\text{O}}_{{2}} {\text{max}}$), followed by two 5-min periods of low intensity (40% theoretical ${\dot{\text{V}}\text{O}}_{{2}} {\text{max}}$) and high intensity (70% theoretical ${\dot{\text{V}}\text{O}}_{{2}} {\text{max}}$). Intensities were calculated using the Åstrand table. Immediately after exercise, participants stopped cycling and rested on the cycle ergometer for 3 min.

Spirometry. The pulmonary function was assessed first under SL and, then, under HH at rest conditions using a spirometer (Easy-on PC; ndd Medical Technologies; Zurich, Switzerland) and its companion software (Connect V03.09.05.19; ndd Medical Technologies; Zurich, Switzerland). This equipment is based on ultrasonic non-contact technology (TrueFlow™), does not require calibration and is independent of environmental variables (humidity, temperature, pressure and changes in composition (molar mass) of air). Participants sitting down in a chair performed a minimum of three valid standard manoeuvres to check test repeatability. The automatic quality control function allows the selection of grade A (optimal) test data. Forced vital capacity (FVC), forced expiratory volume in the first second (FEV1), FEV1/FVC ratio (FEV1/FVC), forced expiratory flow between 25 and 75% of forced vital capacity (FEF25 - 75), peak expiratory flow (PEF), forced expiratory time (FET), forced inspiratory volume capacity (FIVC) and peak inspiratory flow (PIF) were registered. In addition, tidal volume (VT), respiratory frequency (Rf) and lung ventilation (${\dot{\text{V}}\text{E}}$) were measured at HH during the last minute of each exercise intensity.

Oxygen saturation, heart rate and rate of perceived exertion. Oxygen saturation (SaO_2_) was monitored by a pulse oximeter (Nonin Onyx II 9550) from the index finger of the right hand. Heart rate (HR) was obtained with a chest strap sensor (HRM-Dual, Garmin Ltd; Kansas, EUA). The rate of perceived exertion (RPE) was asked according to the Borg scale (Borg 1970). These parameters were recorded during the last minute of each exercise intensity under HH.

Blood pressure and heart rate recovery. Blood pressure (BP) was measured employing a tensiometer (Omron M3) just at the end of the exercise protocol. The HR was recorded at 1, 2 and 3 min after stopping the exercise, while participants rested quietly on the cycle ergometer.

Plantar pressure parameters. Body balance control was evaluated under HH in two conditions, starting with their eyes open (EO) and followed with their eyes closed (EC) by a plantar pressure platform (Podoprint, Namrøl Medical S.L.; Barcelona, Spain). Participants were instructed to rest on the platform barefoot with a relaxed and stable posture. In an EO condition, they had to focus their gaze on a point at 3 m distance and 1.70 m height. After that, it was repeated with EC. Participants maintained both positions for 1 min and rested 1 min between them. Ten postural parameters according to the recorded variation in the centre of pressure (CoP) were evaluated: total track length, track surface, length/surface ratio, mean velocity of CoP, lateral velocity, anterior velocity, lateral variation, anterior variation, *x-axis* displacement and *y-axis* displacement.

Symptoms across the menstrual cycle. CVM- 22 is a self-reported questionnaire validated in the Spanish language, which evaluates the menstrual quality of life. It has 19 questions in three categories: health perception and physical and functional well-being; symptoms; and psychological cognitive well-being. CVM- 22 uses a Likert scale to determine the frequency of each symptom. A score of three points reports a symptom that was “never” experienced, two points “sometimes”, one point “often” and zero point “always”. The higher the score (out of 66), the better is the menstrual quality of life (Torres-Pascual et al. 2019). Moreover, an online survey was created by using Google Forms, to evaluate symptoms across the MC and their negative impact on training and competitions.

### Statistical analysis

Firstly, data distribution was checked with the Shapiro–Wilk test. Cardiorespiratory responses and postural parameters were compared by two-way RM ANOVA, between the MC phases and altitude (SL/HH), exercise intensity (low/high) or eye condition (open/closed). When no normal distribution was found, the Friedman test and Kruskal–Wallis test were performed. For multiple comparisons, post hoc analyses were made according to Tukey and Bonferroni tests. Comparison among MC phases was done with paired *t* test and Wilcoxon test. The Pearson test was used to assess the influence of age or type of sport. Significant differences were considered when *p* ≤ 0.05. Statistical analysis was performed using R (Version 4.4.1).

## Results

The anthropometrical characteristics of the participants and their MC features are represented in Tables 1 and 2, respectively.

### Spirometry

A comparison of resting pulmonary function at SL and HH during the F and L phases by standard spirometry is shown in Table 3. As expected, notable discrepancies between altitudes were observed in FEF25 - 75, PEF and PIF (*p* ≤ 0.0001). These parameters are influenced by the reduction in air density that occurs at high altitudes due to the low barometric pressure causing gas volume expansion (Netzer et al. 2022). No significant discrepancies were identified between the MC phases. Post hoc analysis revealed statistically significant differences between the MC phases and altitude on FEV1/FVC (*p* < 0.0001) and FET (*p* = 0.028).

**Table 3:** Forced spirometry at rest

Parameters	Altitude	Follicular (*n* = 19)	Luteal (*n* = 19)	*p* value MC phase	Cohen’s d	*p* value MC × altitude
FVC (L)	SL	3.97 (0.45)	3.99 (0.46)	0.691	– 0.025	0.223
	HH	3.95 (0.45)	3.92 (0.43)	0.433	0.073	
FEV1 (L)	SL	3.31 (0.43)	3.35 (0.48)	0.260	– 0.083	0.695
	HH	3.35 (0.39)	3.36 (0.43)	0.806	– 0.032	
FEV1/FVC (%)	SL	0.83 (0.06)	0.84 (0.06)	0.841	– 0.09	** < 0.0001**
	HH	0.85 (0.05)	0.86 (0.05)	0.687	– 0.158	
FEF25 - 75 (L/s)	SL	3.67 (0.84)	3.72 (1.03)#	0.545	– 0.058	0.603
	HH	4.09 (1.05)	4.23 (1.11)	0.454	– 0.131	
PEF (L/s)	SL	7.20 (1.63)	7.61 (1.26)#	0.157	– 0.281	0.954
	HH	8.32 (2.00)	8.74 (1.42)	0.200	– 0.247	
FET (s)	SL	6.06 (1.16)	5.84 (1.32)	0.384	0.179	**0.028**
	HH	5.58 (1.35)	5.28 (1.35)	0.314	0.219	
FIVC (L)	SL	3.76 (0.49)	3.76 (0.41)	1.000	– 0.004	0.486
	HH	3.73 (0.49)	3.81 (0.43)	0.095	– 0.18	
PIF (L/s)	SL	4.84 (1.27)	4.79 (1.50)#	0.804	0.035	0.991
	HH	5.61 (1.59)	5.57 (1.52)	0.827	0.029	

Bold character indicates p value lower than 0.05 (*p* < 0.05)

Mean values and standard deviation (SD). # Significant differences between altitudes, SL vs HH (*p* ≤ 0.0001). Interaction between altitude and MC phase factors are in bold for a p value *p* ≤ 0.05

*MC* menstrual cycle, *SL* sea level, *HH* hypobaric hypoxia, *FVC* forced vital capacity, *FEV1* forced expiratory volume in the first second, FEV1/FVC FEV1/FVC Ratio, *FEF25 - 75* forced expiratory flow between 25 and 75% of forced vital capacity, *PEF* peak expiratory flow, *FET* forced expiration time, *FIVC* forced inspiratory volume capacity, *PIF* peak inspiratory flow

### Cardiorespiratory responses during exercise at high altitude

**Fig. 2:**
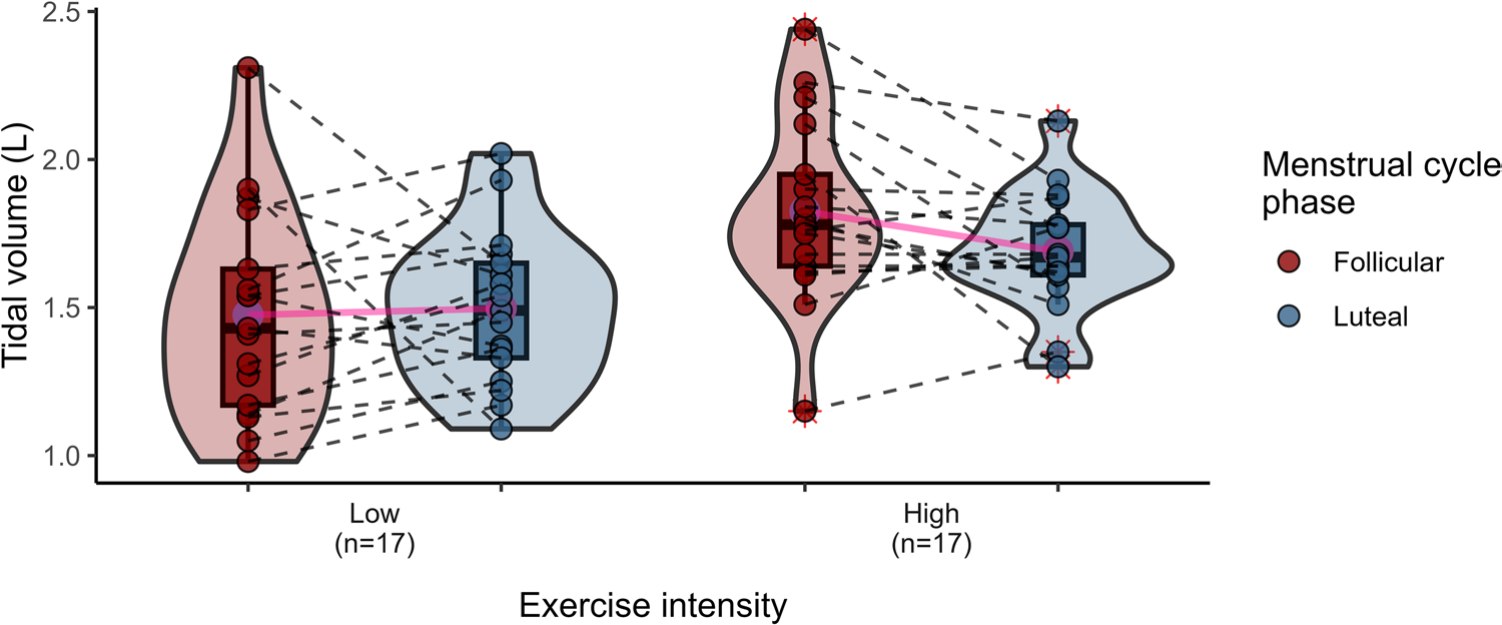
Violin plots illustrating tidal volume (L) according to exercise intensities (low and high) and MC phases (follicular and luteal). The density curves represent the distribution of the data, with inner box plots showing the interquartile range and median values. Individual data points are shown, connected by dashed lines to indicate the repeated measures for each participant. Means are highlighted in red and connected for comparison between phases

**Fig. 3:**
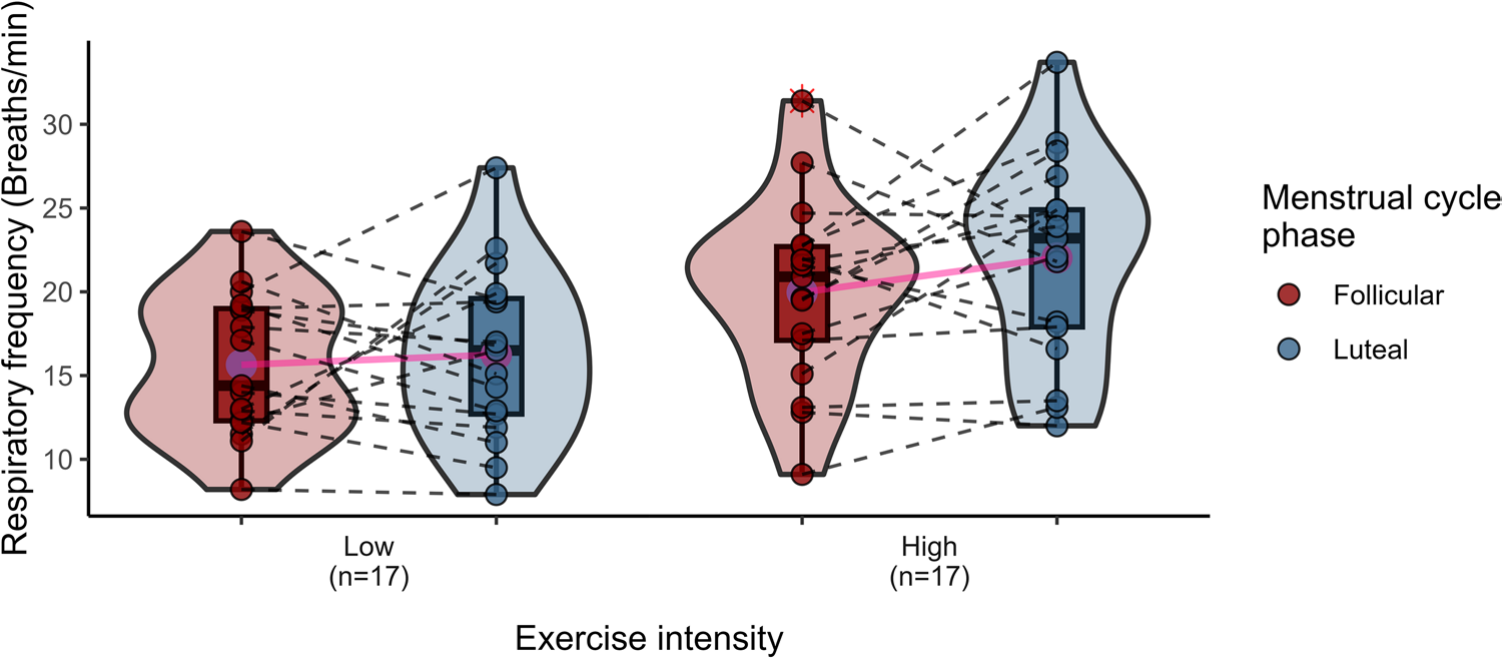
Violin plots illustrating respiratory frequency (breaths/min) according to exercise intensities (low and high) and MC phases (follicular and luteal). The density curves represent the distribution of the data, with inner box plots showing the interquartile range and median values. Individual data points are shown, connected by dashed lines to indicate repeated measures for each participant. Means are highlighted in pink and connected for comparison between phases

**Fig. 4:**
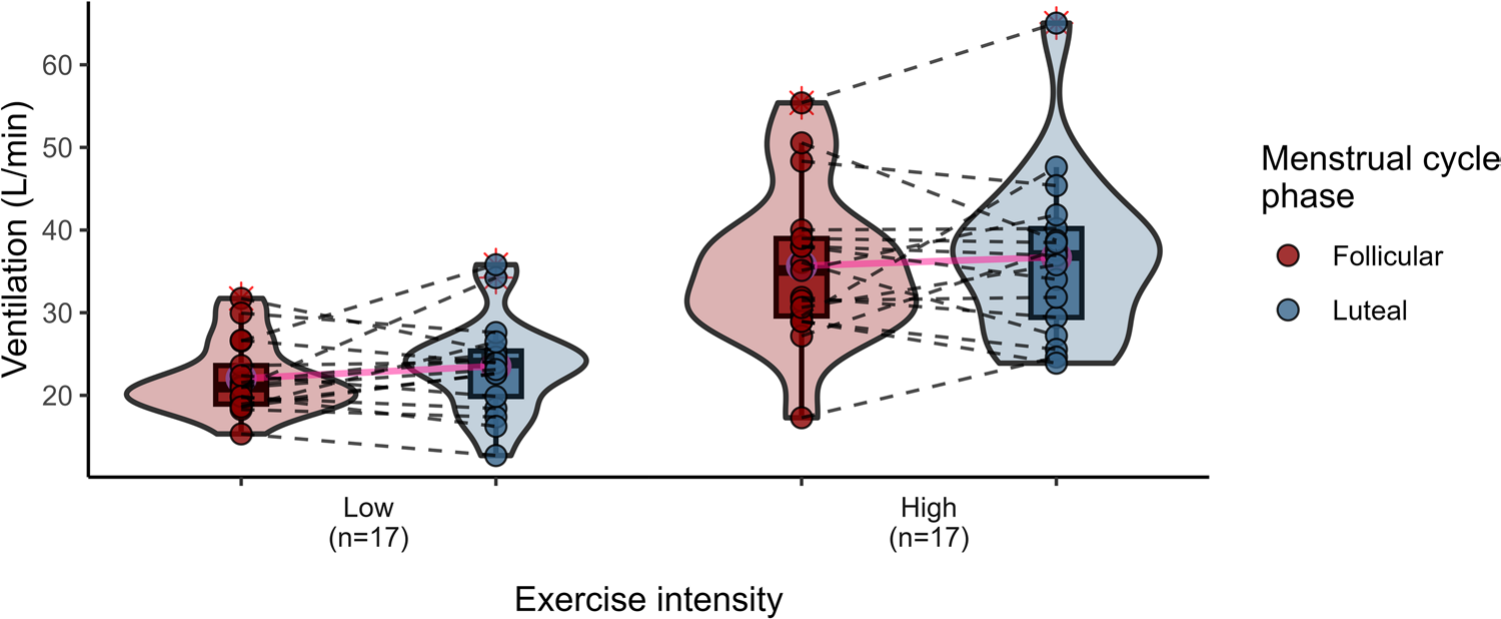
Violin plots illustrating pulmonary ventilation (L/min) according to exercise intensities (low and high) and MC phases (follicular and luteal). The density curves represent the distribution of the data, with inner box plots showing the interquartile range and median values. Individual data points are shown, connected by dashed lines to indicate repeated measures for each participant. Means are highlighted in pink and connected for comparison between phases

**Fig. 5:**
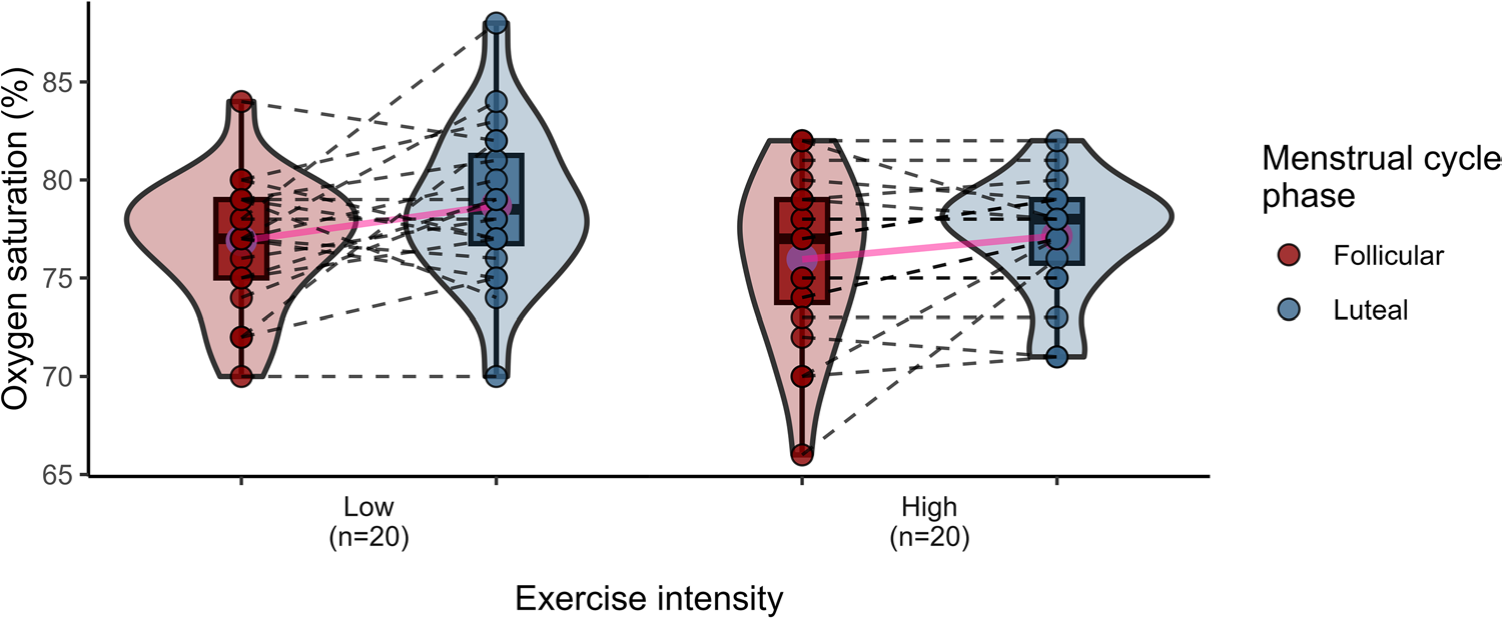
Violin plots illustrating oxygen saturation (%) according to exercise intensities (low and high) and MC phases (follicular and luteal). The density curves represent the distribution of the data, with inner box plots showing the interquartile range and median values. Individual data points are shown, connected by dashed lines to indicate repeated measures for each participant. Means are highlighted in pink and connected for comparison between phases

Cardiorespiratory response data in the F and L phases were obtained from 20 women during a submaximal exercise test at HH (Table 4). Significant differences in SaO_2_ (*p* ≤ 0.01) were observed between the two phases of the MC, with higher oxygenation in the L (vs. F) phase, regardless of the intensity of exercise. Additionally, VT recorded at high intensity was significantly reduced in the L compared to the F phase (*p* = 0.053), resulting in a 7.69% decline (Fig. 2). The general trend, not always statistically significant, of Rf, V̇E and SaO_2_, revealed an increase in the L phase (vs. F), both at low and high intensity (Figs. 3, 4 and 5) This pattern, is consistent with a higher work of breathing in females to achieve the ${\dot{\text{V}}\text{E}}$ demands during exercise (Guenette et al. 2007). Multiple comparisons revealed statistically significant differences in VT, ${\dot{\text{V}}\text{E}}$ and Borg between the intensity and MC phases (*p* = 0.033, *p* ≤ 0.0001, *p* ≤ 0.0001, respectively). There were also significant pair comparisons between exercise intensities at the same and different MC phases. This seems to suggest that the intensity of exercise plays a significant role in the influence of female hormones at high altitudes.

**Table 4:** Cardiorespiratory responses at different exercise intensities under hypobaric hypoxia

Parameters	Exercise intensity	Follicular (*n* = 17)	Luteal (*n* = 17)	*p* value MC phase	Cohen’s d	*p* value MC phase × exercise intensity
VT (L)	Low	1.47 (0.36)	1.50 (0.26)#	0.786	– 0.073	**0.033**
	High	1.82 (0.31)	1.69 (0.20)*	0.053	0.506	
Rf (breaths/min)	Low	15.64 (4.21)	16.25 (5.13)#	0.649	– 0.131	0.223
	High	19.97 (5.56)	22.03 (6.07)	0.154	– 0.353	
V̇E (L/min)	Low	22.05 (4.38)	23.54 (5.85)#	0.353	– 0.289	** < 0.0001**
	High	35.67 (9.38)	36.72 (10.16)	0.818	– 0.109	
SaO_2_ (%)	Low	76.90 (3.26)	78.70 (4.00) +	0.061	– 0.484	0.625
	High	75.95 (4.29)	77.15 (2.96)	0.090	– 0.326	
HR (bpm)	Low	122.95 (11.33)	123.90 (13.36)#	0.704	– 0.078	0.579
	High	146.30 (14.02)	148.30 (13.53)	0.203	– 0.147	
Borg scale	Low	9.00 (1.49)	9.00 (1.59)#	1.000	0	** < 0.0001**
	High	12.45 (1.05)	11.95 (1.54)	0.175	0.378	

Bold character indicates p value lower than 0.05 (*p* < 0.05)

Mean values and standard deviation (SD). *Significant differences between MC phases (*p* ≤ 0.05), # significant differences between exercise intensity at simulated altitude, low vs high (*p* ≤ 0.0001), + significant differences between the MC phases not taking into account the exercise intensity (*p* ≤ 0.01). Interaction between exercise intensity and MC phase factors are in bold for a *p* value *p* ≤ 0.05

*MC*: menstrual cycle, *VT* tidal volume, *Rf* respiratory frequency, ${\dot{\text{V}}\text{E}}$ minute ventilation, *SaO*_*2*_ peripheral arterial oxygen saturation, *HR* heart rate

### Blood pressure and heart rate recovery after exercise

The systolic and diastolic pressure were measured immediately after the end of the exercise and no differences between the MC phases were found. Moreover, HR recovery was measured on the 3-min post-exercise and no significant differences were observed between the MC phases.

### Body balance control

Postural control values were assessed at simulated HH, according to the CoP using a foot pressure platform in stable posture with EO and EC. As demonstrated in Table 5, only lateral velocity at EO was statistically different between the MC phases, higher in the L (4.88) vs. F (4.24) phase (*p* = 0.045). However, significant differences between eye conditions were found in CoP track length, mean velocity of CoP and anterior velocity (*p* ≤ 0.05), curiously with higher unbalances on the EO condition. Interaction analysis showed significant global effects on CoP track length, mean velocity of CoP and lateral and anterior velocities (*p* = 0.033, *p* = 0.049, *p* = 0.028, *p* = 0.029, respectively). A subsequent analysis of the pairwise comparisons revealed significance on the CoP track length, mean velocity of CoP and lateral velocity, indicating more instability on the L phase in the EO condition (vs. F with EC).

**Table 5:** Balance results under hypoxia

Parameters	Eyes	Follicular (*n* = 19)	Luteal (*n* = 19)	*p* value MC phase	Cohen’s d	*p* value MC phase × eyes
CoP track length (mm)	Open	313.01 (90.06)	350.36 (95.52)#	0.123	– 0.400	**0.033**
	Closed	280.32 (77.53)	299.08 (91.43)	0.418	– 0.223	
Surface (mm^2^)	Open	179.68 (183.46)	158.36 (165.03)	0.595	0.124	0.837
	Closed	114.24 (109.23)	139.13 (189.80)	0.709	– 0.162	
Length/surface (mm^−1^)	Open	4.84 (5.05)	4.49 (3.37)	0.952	0.082	0.846
	Closed	5.03 (4.93)	5.44 (4.54)	0.702	– 0.087	
Mean velocity of CoP (mm/s)	Open	5.38 (1.51)	6.05 (1.66)#	0.165	– 0.417	**0.049**
	Closed	4.87 (1.35)	5.19 (1.56)	0.421	– 0.222	
Lateral velocity (mm/s)	Open	4.24 (1.11)	4.88 (1.43)*	0.045	– 0.488	**0.028**
	Closed	3.88 (1.17)	4.15 (1.12)	0.408	– 0.232	
Antero-posterior velocity (mm/s)	Open	3.26 (1.17)	3.49 (1.04)#	0.360	– 0.211	**0.029**
	Closed	2.88 (0.86)	3.08 (1.25)	0.587	– 0.193	
Lateral variation (mm)	Open	2.03 (1.18)	1.78 (0.86)	0.632	0.236	0.669
	Closed	1.87 (1.05)	1.63 (0.82)	0.184	0.259	
Antero-posterior variation (mm)	Open	3.41 (2.74)	3.60 (2.56)	0.763	– 0.072	0.832
	Closed	3.23 (2.13)	3.30 (2.70)	0.984	– 0.031	
*x-axis* displacement (mm)	Open	– 9.16 (8.88)	– 9.19 (10.59)	0.393	0.003	0.398
	Closed	– 7.39 (8.92)	– 6.88 (9.80)	0.745	– 0.055	
*y-axis* displacement (mm)	Open	2.56 (20.81)	– 1.23 (12.75)	0.745	0.221	0.229
	Closed	0.54 (16.78)	6.92 (14.13)	0.151	– 0.408	

Bold character indicates p value lower than 0.05 (*p* < 0.05)

Mean values and standard deviation (SD). *Significant differences between the MC phases (*p* ≤ 0.05), # significant differences between eyes’ condition, EO vs EC (*p* ≤ 0.0001). Interaction between eyes’ condition and MC phase factors are in bold for a *p* value *p* ≤ 0.05

*MC* menstrual cycle, *EO* open eyes, *EC* eyes closed, *CoP* centre of pressure

### Menstrual cycle symptoms and impact on exercise performance

**Fig. 6:**
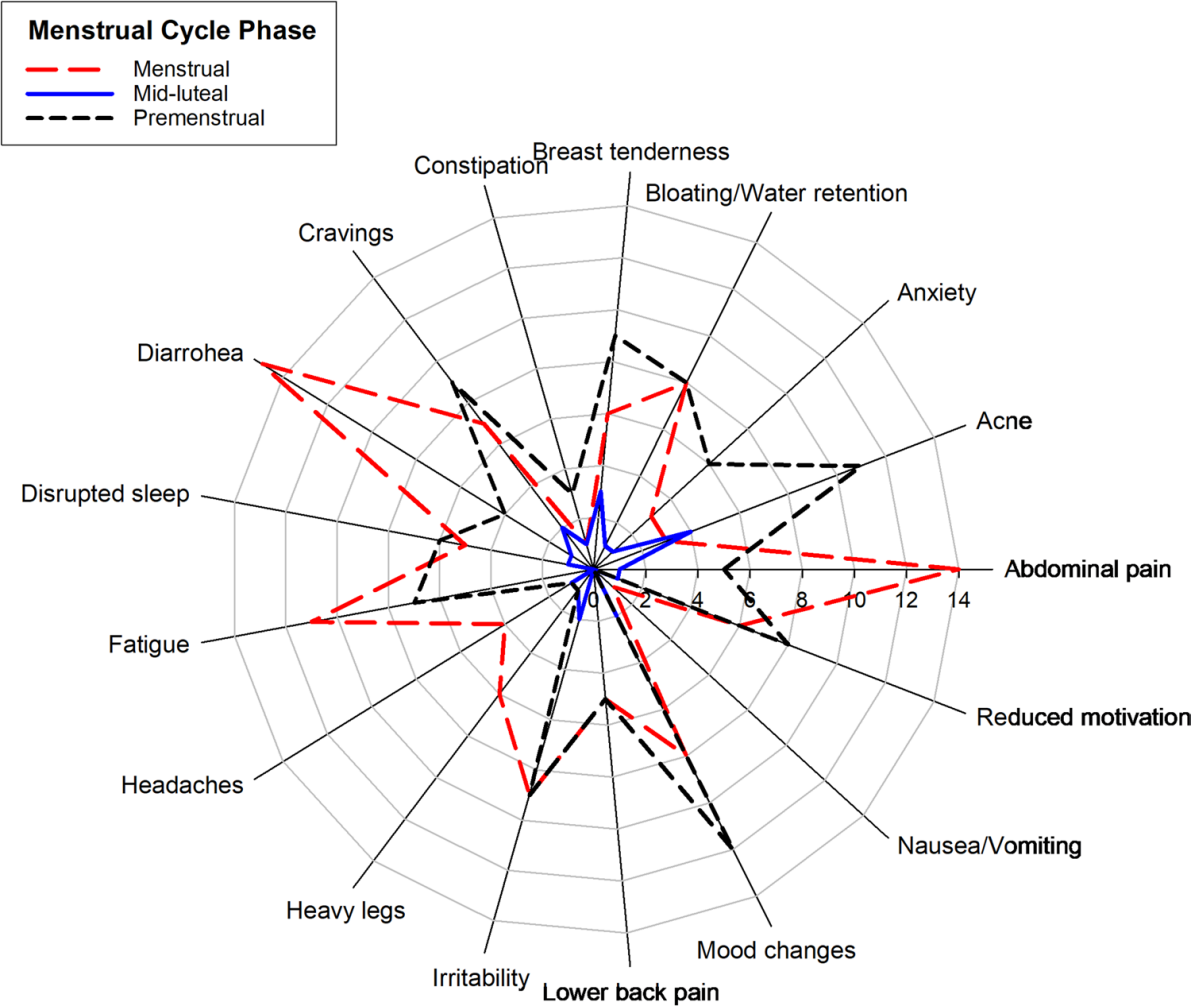
Radar plot showing the distribution of symptoms across the MC phases (menstrual, mid-luteal and premenstrual). The axes represent different symptoms. Each line corresponds to a different phase: dashed black lines for menstrual, solid red lines for mid-luteal and dotted blue lines for premenstrual

**Fig. 7:**
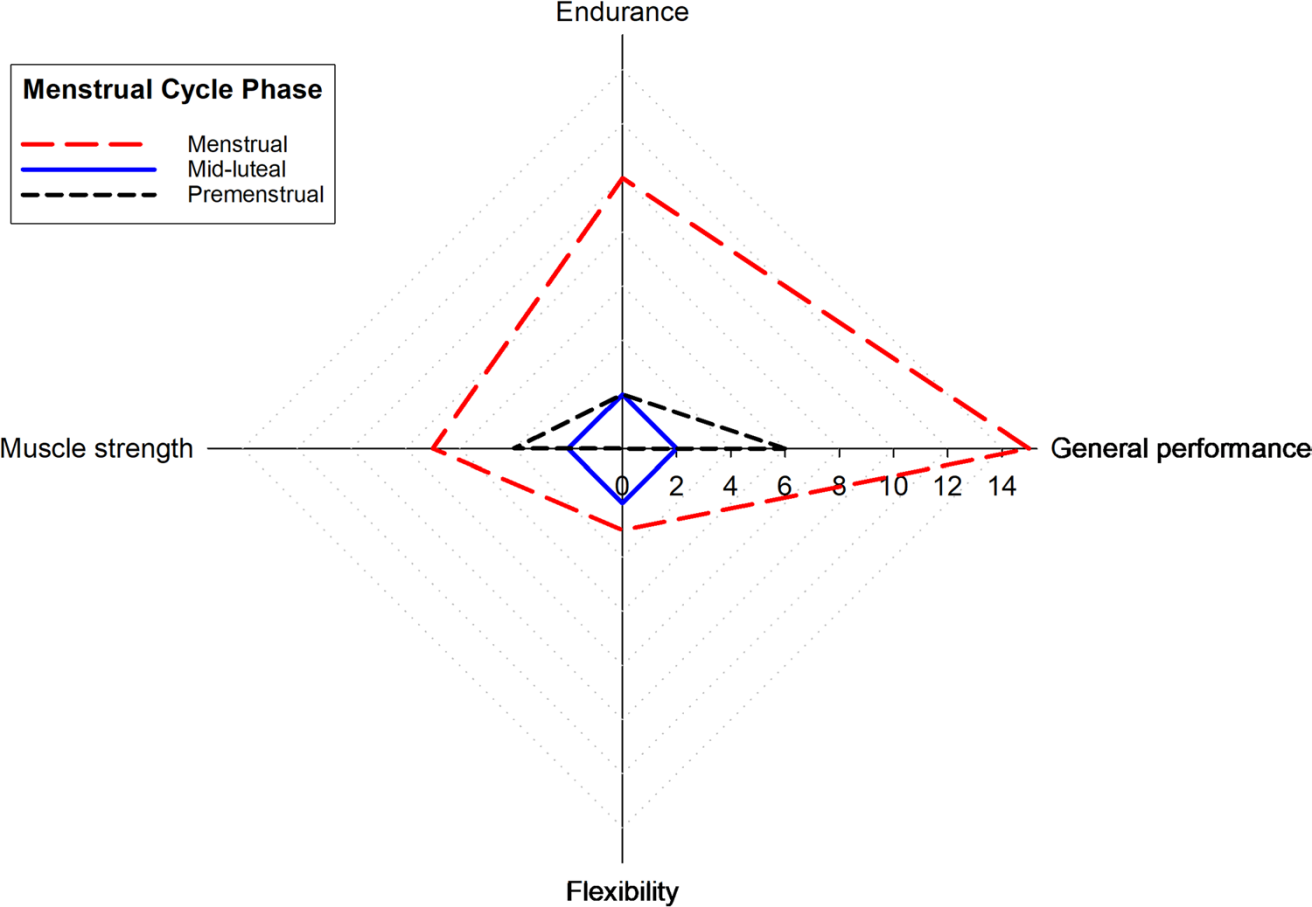
Radar plot comparing the performance metrics across the MC phases (menstrual, mid-luteal and premenstrual). The axes represent muscle strength, endurance, flexibility and overall performance. Each line corresponds to a different phase, with dashed black lines for menstrual, solid red lines for mid-luteal and dotted blue lines for premenstrual

Menstrual quality of life was assessed by the CVM- 22 questionnaire and surveyed participants online to measure the negative impact of MC phases on training and competition. The CVM- 22 results (Table 6), clearly indicated that psychological and cognitive well-being were the most affected areas during the menstrual days. Participants’ “sometimes” responses, in psychological well-being, physical health and symptoms, were 47.50, 39 and 27.50%, respectively. Frequencies of symptoms and the negative impact on exercise performance are shown in Figs. 6 and 7. The premenstrual phase was the most symptomatic phase, followed closely by the menstrual phase, with an average of 6.12 and 5.89 symptoms, respectively. Mood changes, acne, cravings and irritability were more prevalent in the premenstrual phase, characterised in the PMS (Daley 2009). The menstrual phase was more affected by somatic symptoms than mental ones. This result contrasts with the answers of CVM- 22. Menstrual days had the greatest impact on general performance, with 88.2% (n = 15) of respondents reporting negative effects. This was followed by premenstrual days with 35.3% (*n* = 6). These results align with many respondents who altered their training load during the menstrual days (80%, *n* = 16). Moreover, 40% (*n* = 8) of participants interrupted their training routine during this MC phase.

**Table 6:** Results of CVM- 22 questionnaire (*n* = 20)

Dimensions	Frequencies			
	Always	Often	Sometimes	Never
Health perception and physical/functional well-being	5	13	39	43
Psychological and cognitive well-being	5.63	16.88	47.50	30
Symptoms	3.75	15	27.50	53.75

Frequencies are expressed as a percentage of the total sample. *CVM- 22* Menstrual Quality of Life Questionnaire

## Discussion

This study aimed to evaluate the effects of MC on aerobic performance under HH. To determine the exercise performance, ventilatory and cardiorespiratory responses under equal intensity exercise and postural control were assessed on two MC phases: F and L (Elliott-Sale et al. 2021). Our key findings included a significantly decreased VT at high intensity in L compared to F and increased SaO_2_ in L compared to F during exercise at a simulated altitude of 4000 m. Although Rf and V̇E varied, as expected, with increased levels in the L phase, results were not statistically significant. Furthermore, higher lateral velocities were found during the L phase only with EO. Therefore, our findings do not confirm that physical performance is altered by the MC phase.

### Spirometry

Forced spirometry parameters based on anatomical features remained consistent throughout the cycle phases, both at SL and HH (Table 3), in agreement with a previous study that found no differences in the MC phases on FVC and FEV1 (Beidleman et al. 1999). On the contrary, other studies found increased PEF rate in L (vs. F) (Samsudeen and Rajagopalan 2016) and a positive correlation between FVC, FEV1 and FEV1/FVC (Mannan et al. 2008) with plasma progesterone in normoxia. As expected, significant differences were found between altitudes on FEF 25–75, PEF, and PIF (*p* ≤ 0.0001), because of the lower air density at the low barometric pressure of HH, where higher values were found because of reduced resistance to air convection (Netzer et al. 2022). Furthermore, a comprehensive evaluation of the relationship between the MC phases and altitudes on FEV1/FVC revealed a statistically significant association (*p* = 0.0001). Specifically, this variable exhibited higher levels in L at HH (as compared to L and F at SL) and in F at HH (as compared to F at SL). Furthermore, FET was reduced in the L phase at HH compared with SL (*p* = 0.016).

### Cardiorespiratory responses during exercise at high altitude

No significant changes were detected between the MC phases on Rf, ${\dot{\text{V}}\text{E}}$, SaO_2_, HR and Borg under HH (Table 4) at the two exercise intensities. These findings are in agreement with previous reports by other authors (Dombovy et al. 1987; Beidleman et al. 1999; Takase et al. 2002; MacNutt et al. 2012; Citherlet et al. 2024a) and also at resting conditions (MacNutt et al. 2012; Citherlet et al. 2024a). Conflicting findings were found with higher ${\dot{\text{V}}\text{E}}$ in the L vs. the F phases during exercise at 3600 m (Brutsaert et al. 2002) and SL (Rael et al. 2021). The findings of this study contradict the results of previous research on resting V̇E under hypobaric hypoxia (Tagliapietra et al. 2024), but are consistent with those in normobaric hypoxia (Citherlet et al. 2024b). Several studies (Basualto-Alarcón et al. 2012; Faiss et al. 2013; Beidleman et al. 2014) have demonstrated that, under hypobaric conditions, the respiratory and cardiovascular systems do not respond in the same way as under normobaric hypoxia. This aspect has been extensively discussed (Millet and Debevec 2020; Richalet 2020). Despite the limitations of extrapolating normobaric conditions to hypobaric conditions, owing to disparities in arterial PCO_2_ and plasma pH, further research is necessary to provide a more comprehensive understanding of the HVR in females. Moreover, a higher rating of perceived exertion during menses was reported as compared to other phases (Delp et al. 2023). The hypothesis of increased aerobic performance in the L phase was based on previous studies, conducted at SL, which found higher ventilation because of the effects of progesterone in this phase that enhanced the chemosensitivity of the hypothalamus chemoreceptors, lowering the threshold of the medullary respiratory centre and thus increasing ventilation (Williams and Krahenbuhl 1997; Janse de Jonge 2003; Samsudeen and Rajagopalan 2016; Goldsmith and Glaister 2020; Rael et al. 2021). This effect is enhanced by oestradiol, which is found during the mid-luteal days, increasing the number of progesterone receptors and stimulating the ${\dot{\text{V}}\text{E}}$ (Brodeur et al. 1986). Moreover, significant interactions of MC phases and exercise intensity were found on VT, ${\dot{\text{V}}\text{E}}$ and Borg (*p* = 0.033, < 0.0001, < 0.0001, respectively).

VT during high-intensity exercise at HH was significantly lower in the L phase (1.69 L) than in the F (1.82 L) (*p* = 0.053) with a moderate effect size (Cohen’s d = 0.506), as shown in Fig. 2. Another study found a decrease in resting VT in the L than in the F phases (0.71 and 0.81 L; *p* = 0.027, respectively), although these results were no longer evident during exercise at high altitude (Tagliapietra et al. 2024). Contrary to this trend, Rf and ${\dot{\text{V}}\text{E}}$ increased in the L phase (Figs. 3 and 4), although the results were not statistically significant with an almost moderate size effect of Rf at high altitude (Cohen’s d = 0.353). These findings showed the respiratory female pattern with greater breathing work to compensate for the ventilation demands during exercise, probably caused by their smaller lung volumes and diameter airways (Guenette et al. 2007).

SaO_2_ increased during the L compared to the F phase at HH independently of the exercise intensity (*p* ≤ 0.01), with marginally significant results (*p* = 0.06 and *p* = 0.090, for low and high intensity, respectively) and moderate effect sizes at low intensity (Cohen’s d = 0.484) and high intensity (Cohen’s d = 0.326), as can be seen in Fig. 5. This finding is supported by other authors (Beidleman et al. 1999; Richalet et al. 2020). Surprisingly, SaO_2_ increased independently of the ventilation, as was also reported previously in acute altitude exposure (Bender et al. 1989; Beidleman et al. 1999). An improvement of arterial O_2_ content and transport under acute hypoxia (Beidleman et al. 1999) and an increase in pulmonary diffusion capacity (Smith et al. 2015) during the L phase could explain these results. In contrast, no changes were also found in other reports (Dombovy et al. 1987; MacNutt et al. 2012). Moreover, other authors found higher SaO_2_ levels in the mid-follicular phase during exercise, but no changes at rest (Citherlet et al. 2024a).

In summary, there was no significant and systematic variation in HVR across the MC. However, it is noteworthy that female physiological particularities may exert a minor effect on respiratory function, particularly concerning VT and SaO_2_, although this effect is insufficient to impact aerobic performance (Beidleman et al. 1999). Discrepant findings between studies could be due to differences in the characteristics of the participants, sample size, type and level of hypoxia, acclimatisation status, MC determination and exercise intensities.

### Blood pressure and heart rate recovery after exercise

Systolic and diastolic pressure and HR during recovery from exercise did not change significantly across the MC under HH. Similar findings have been reported at 4300 m at rest (Mazzeo et al. 1998), however, values were reported on day 2 after arrival to altitude, in contrast to this study, which was under acute hypoxia exposure. Inconsistent results have also been found at rest in normoxia (Thakrar et al. 2024; Citherlet et al. 2024a). Opposite effects on vascular tone, vasodilatory effects by oestrogens and vasoconstrictor effects by progesterone (Sarrel 1999), could explain the variable results in the literature. In agreement with a previous report (Tagliapietra et al. 2024), HR recovery rate has not changed across the MC.

### Body balance control

The lateral velocity of CoP at EO increased significantly during the L phase with a moderate size effect (*p* = 0.045; Cohen’s d = 0.488), which suggests more instability in this phase compared to the F phase (Table 5). To our knowledge, this is the first report to study the effects of MC on body balance under hypoxic conditions. Previous studies at SL are in agreement with our results showing a similar tendency with greater unbalance during the mid-luteal phase (*p* = 0.06) (Fridén et al. 2003), while others found higher lateral and anterior velocity during the ovulation phase (Lee et al. 2017; Aziz et al. 2023) and pre-ovulation period (Elvan et al. 2024). Oestrogen and progesterone receptors located in the skeletal muscle, bone, ligament and nervous system could change the structure and function of those tissues and be related to the changes in postural control and balance across the MC (Lee et al. 2017). Length, the mean velocity of CoP and anterior velocity at EO did not change between the MC phases, although moderate and small effects were found (Cohen’s d = 0.400, 0.417 and 0.211, respectively). This trend was also supported by other reports (Reschechtko et al. 2023; Aziz et al. 2023).

Although the influence of vestibular, visual and somatosensory systems on body balance and postural control has been well described (Hall 2015; Rodrigues et al. 2017), surprisingly, our results presented a greater instability of the participants with EO, independently of the MC. It has been hypothesised that HH rapidly alters visual function, affecting the central nervous system by increasing the reaction time (Nordahl et al. 1998; Pun et al. 2018; Debenham et al. 2021) and leading to remarkable cognitive limitations (Regard et al. 1989). These alterations could justify a more unstable control of body balance with EO than EC under acute hypoxia, since at SL, the role of vision determined a better static postural balance (Tomomitsu et al. 2013), also among athletes (Hammami et al. 2014). However, there is no consistent explanation of how hypoxia can affect the sensory–motor pathways and impact neural balance control (Debenham et al. 2021).

Interactions between MC phases and eyes’ condition were found on CoP track length, mean velocity of CoP, and lateral and anterior velocities (*p* = 0.033, 0.049, 0.028, 0.029, respectively). Post hoc analysis revealed a reduction in postural stability during the L phase with EO (vs. F with EC) on CoP track length, the mean velocity of CoP and the lateral velocity, which aligns with previous reports (Fridén et al. 2005). Further research is required to ascertain the true implications of the menstrual cycle and female sexual hormones on body balance control in the context of acute hypoxia and visual function.

### Menstrual cycle symptoms and impact on exercise performance

In our study, 18 out of 20 participants reported symptoms during the MC. The most common symptoms declared, without taking into account the phase, were abdominal pain, mood changes, diarrhoea and irritability. The mean of symptoms per menstrual cycle was 7.093. According to the phases, the premenstrual phase was the most affected with an average of 6.12 symptoms, followed by the menstrual phase with a mean of 5.89 symptoms and the mid-luteal phase with 2.75 symptoms.

During the menstrual phase, we found the most prevalent symptoms to be diarrhoea (75%, *n* = 15), abdominal pain (70%, *n* = 14) and fatigue (55%, *n* = 11), which is in line with the literature (Solli et al. 2020; Oester et al. 2024), followed by mental symptoms such as irritability (45%, *n* = 9) and mood changes (40%, *n* = 8), while other authors suggested that mental affectations are more frequent during the menstrual phase than somatic symptoms (Bruinvels et al. 2021). During the premenstrual phase, mood changes were the most prevalent symptoms (60%, *n* = 12), followed by acne, cravings, irritability, reduced motivation and bloating (Fig. 6).

In this study, 90% (*n* = 18) of the participants reported symptoms negatively affecting their sports performance. This finding is supported by the results of a recent extensive scoping review, which found between 2.8% and 100% of the athletes reported that the main reason for their perceived impact on performance was the menstrual cycle symptoms (Oester et al. 2024). The menstrual phase days were the most affected with 88.2% (*n* = 15), which aligns with previous studies (Martin et al. 2018; Solli et al. 2020; Doohan et al. 2023; McNulty et al. 2023; Taim et al. 2024) and followed by premenstrual phase days that affect negatively their performance in 35.3% (*n* = 6), while it has seen a higher prevalence (76.6%) associated with premenstrual syndrome and decreased performance (Yi and Bae 2021). This study used the term “premenstrual symptoms” to consider the affectations between one to four last days before menstruation. It should be noted that there is no consensus in the literature on the terminology used to discuss symptoms before and during menses, the most common terms being premenstrual syndrome or premenstrual symptoms (Angst et al. 2001; Oester et al. 2024). Up to 11.8% of our participants reported that the mid-luteal phase affected their performance. Likewise, it has been shown how phase 3 and phase 2 of the cycle were the least negatively affected physical activity and performance days of the cycle (Solli et al. 2020; Ekenros et al. 2022). Endurance exercise was the physical quality most affected during menstruation (83.3%, *n* = 10), followed by muscle strength (63.6%, *n* = 7), although these answers could be biased due to the participants being endurance athletes (Fig. 7). In contrast to our study, it has been reported that muscle strength was impacted to the same extent as aerobic fitness (Ekenros et al. 2022).

A significant number of respondents (80%, *n* = 16) reported having changed their training sessions by adjusting their intensity or duration due to the symptoms, and these data are consistent with the numbers of athletes who reported symptoms during the menstrual cycle (*n* = 18). Similarly, other reports described that parts of training during the menstrual and premenstrual phases were modified due to the symptoms (Righi and Barroso 2022; Prado et al. 2022), but in contrast, in another report, only 22% of the athletes reported adapting their training due to menstrual cycle side effects, even though they reported a high incidence of abdominal pain (83%) (Solli et al. 2020). In our study, the proportion of athletes who refrain from training or competition on account of the symptoms was 40% (n = 8); this trend has some concordance with previous reports (Ekenros et al. 2022; McNulty et al. 2023) and contrasts with other findings (Martin et al. 2018), which reported that only 4.2% of the female athletes opted out of training or competition because of menstruation.

Despite the high number of symptoms reported in the literature across the MC, there is no consistency in the number of participants who adapt or miss training sessions. This discrepancy could be explained by different hypotheses. Probably, while female athletes can report menstrual-related symptoms, they may not view these as a significant reason to stop their training. Additionally, they seem reluctant to discuss their symptoms with their coaches (Findlay et al. 2020). Effective communication between athletes and coaches is essential to facilitate open dialogue about menstrual health and related symptoms, challenging the stigma surrounding this topic (Righi and Barroso 2022). There is a general agreement that more research is needed to delve into the relationship between symptoms and performance throughout the menstrual cycle, mainly due to the wide methodological differences found in the literature.

Almost half of the participants (47.5%) responded to the CVM- 22 questionnaire that psychological symptoms (irritability, mood changes, sadness, etc.) are present “sometimes” during their menstrual phase, followed by physical health (39%) and symptoms (27.50%) (Table 6), which does not agree with the results of the online form that showed physical symptoms as the most prevalent during the menstrual phase.

## Conclusions

In summary, despite significant VT at high exercise intensity and greater SaO_2_ during exercise in the L phase, as well as a potential decrease in balance in the L phase, these results suggest that hormonal fluctuation across the MC does not markedly impact exercise performance under acute HH exposure. However, a significant interaction between MC and exercise intensity on certain cardiorespiratory parameters underpins how training load must be adjusted when female athletes train, especially under hypoxic conditions. Somatic symptoms are the most common during the menstrual days, followed by the pre-menstrual phase and 80% of our participants usually alter their training to deal with MC symptoms. These findings may have important implications for women who work or participate in athletic competitions or recreational activities involving short-term exposure to moderate or high altitudes. Further research is needed to corroborate our findings and contrast them with those from healthy sedentary women to verify how trained status might affect physiological responses to exercise and physical capacity throughout the menstrual cycle.

## Data Availability

The data supporting the findings of this study are available from the corresponding author upon reasonable request.

## Abbreviations

BP: Blood pressure
CoP: Centre of pressure
CVM- 22: Menstrual Quality of Life Questionnaire
EC: Eyes closed
EO: Eyes open
F: Early follicular phase
FEF25 - 75: Forced expiratory flow between 25 and 75% of forced vital capacity
FET: Forced expiratory time
FEV1: Forced expiratory volume in the first second
FEV1/FVC: Forced expiratory volume in the first second/forced vital capacity ratio
FIVC: Forced inspiratory volume capacity
FVC: Forced vital capacity
HCR: Hypoxic cardiac response
HH: Hypobaric hypoxia
HR: Heart rate
HVR: Hypoxic ventilatory response
L: Mid-luteal phase
MC: Menstrual cycle
PEF: Peak expiratory flow
PIF: Peak inspiratory flow
Rf: Respiratory frequency
RPE: Rate of perceived exertion
SaO_2_: Arterial oxygen saturation
SL: Sea level
$${\dot{\text{V}}\text{E}}$$: Ventilation
${\dot{\text{V}}\text{O}}_{{2}} {\text{max}}$: Maximal rate of oxygen consumption
VT: Tidal volume
